# Edge-Guided Camouflaged Object Detection via Multi-Level Feature Integration

**DOI:** 10.3390/s23135789

**Published:** 2023-06-21

**Authors:** Kangwei Liu, Tianchi Qiu, Yinfeng Yu, Songlin Li, Xiuhong Li

**Affiliations:** Key Laboratory of Signal Detection and Processing, Department of Information Science and Engineering, Xinjiang University, Urumqi 830017, China; lkw21@stu.xju.edu.cn (K.L.);

**Keywords:** camouflaged object detection, multi-level feature integration, attention mechanism, boundary semantic information

## Abstract

Camouflaged object detection (COD) aims to segment those camouflaged objects that blend perfectly into their surroundings. Due to the low boundary contrast between camouflaged objects and their surroundings, their detection poses a significant challenge. Despite the numerous excellent camouflaged object detection methods developed in recent years, issues such as boundary refinement and multi-level feature extraction and fusion still need further exploration. In this paper, we propose a novel multi-level feature integration network (MFNet) for camouflaged object detection. Firstly, we design an edge guidance module (EGM) to improve the COD performance by providing additional boundary semantic information by combining high-level semantic information and low-level spatial details to model the edges of camouflaged objects. Additionally, we propose a multi-level feature integration module (MFIM), which leverages the fine local information of low-level features and the rich global information of high-level features in adjacent three-level features to provide a supplementary feature representation for the current-level features, effectively integrating the full context semantic information. Finally, we propose a context aggregation refinement module (CARM) to efficiently aggregate and refine the cross-level features to obtain clear prediction maps. Our extensive experiments on three benchmark datasets show that the MFNet model is an effective COD model and outperforms other state-of-the-art models in all four evaluation metrics ($S_{\alpha }$, $E_{\phi }$, $F_{\beta }^{w}$, and MAE).

## 1. Introduction

In nature, organisms use body color, texture, and coverings to conceal themselves within their surroundings, thereby avoiding detection by predators. Camouflaged object detection (COD) is an emerging computer vision segmentation task that aims to segment these objects that blend perfectly into their surroundings [1]. Unlike salient object detection [2,3,4,5], which segments salient objects with distinct boundaries and high contrast with their backgrounds, camouflaged objects often lack clear visual boundaries with their backgrounds and may be obscured by other objects in their surroundings, which makes accurate camouflaged object detection more challenging. Nevertheless, due to its significant research implications and wide application in medical image processing (e.g., polyp segmentation [6], lung infection segmentation [7]), pest detection [8], defect detection [9], and underwater object detection [10], COD has become a research hotspot.

Traditional camouflaged object detection methods [11,12,13,14,15] typically rely on hand-crafted features (textures, colors, edges, etc.) to differentiate camouflaged objects from their surroundings. However, due to their limited ability to extract and analyze high-level semantic information, these methods tend to perform poorly in challenging COD scenarios. In contrast, in recent years, benefiting from the development of deep-learning-based methods, Fan et al. [1] made a substantial contribution to this field by comprehensively studying the COD task and creating the COD10K dataset. They proposed a search and recognition network called SINet. This network uses the simulation of a predator’s predation process in nature to decouple COD into two processes, rough localization and accurate segmentation, thereby achieving the precise segmentation of camouflaged objects. Subsequently, a series of works [16,17,18,19,20,21] have further explored this area.

While the existing methods achieve decent detection performance in most scenarios, there is still considerable room for improvement when dealing with highly challenging situations. In the COD task, multi-level feature fusion also plays a vital role [22]. Convolutional neural networks (CNNs) are known to extract low-level image features through the initial layers. As the network further deepens and processes these low-level features, it effectively captures and incorporates rich semantic information into higher-level features. Additionally, some methods [1,21,23] tend to prioritize the localization of the camouflaged object, overlooking the significance of edge refinement.

In response to the aforementioned issues, we propose a general framework for COD called the multi-level feature integration network (MFNet), which focuses on learning and integrating multi-level contextual features from input images. Specifically, we introduce the edge guidance module (EGM), which generates the edges of camouflaged objects by combining high-level semantic information and low-level spatial details, and guides the network to obtain a more explicit depiction of the edges of camouflaged objects. In addition, we design a multi-level feature integration module (MFIM) to extract and integrate high-level semantic and spatial details in multi-level features. To efficiently aggregate and refine cross-level contextual information, we propose a context aggregation refinement module (CARM) to filter interference information via the attention mechanism [24] and capture and refine multi-scale contextual information via atrous convolution and asymmetric convolution. Our proposed method can significantly improve the detection performance compared to the state-of-the-art methods. The main contributions of this paper are four-fold:

- We propose the novel MFNet to investigate the effectiveness of adjacent layer feature integration in the COD task and confirm the rationality of fully capturing contextual information through the interaction of adjacent layer features;
- We propose an edge guidance module to explicitly learn object edge representations and guide the model to discriminate camouflaged objects’ edges effectively;
- We propose a multi-level feature integration module to efficiently extract and integrate global semantic information and local detail information in multi-scale features;
- We propose a context aggregation refinement module to aggregate and refine cross-layer features via the attention mechanism, atrous convolution, and asymmetric convolution.

## 2. Related Work

### 2.1. Camouflaged Object Detection

Traditional COD methods segment camouflaged objects by extracting artificial features such as texture, color, edge, contrast, and 3D convexity in camouflage scenes [11,12,13,14,15]. However, these features are inefficient in complex scenes.

With the introduction of the large-scale COD dataset COD10K and the animal-predation-inspired baseline SINet by Fan et al. [1], more and more deep-learning-based COD methods have emerged. For instance, Mei et al. [21] proposed PFNet, which uses high-level semantic information to roughly localize camouflaged objects and then wipe out the false positive and false negative areas, which can distract the segmentation results. Zhai et al. [18] proposed MGL, which models the localization and refinement process in camouflaged object detection through graph convolutional networks. Li et al. [25] proposed JCSOD, which employs a joint adversarial learning framework to perform both salient object detection (SOD) and COD tasks to improve the accuracy and robustness of camouflaged object detection. Yang et al. [19] proposed UGTR, a method that combines a convolutional neural network and Transformer, to leverage a probabilistic representation model to learn the uncertainty of camouflaged objects within the Transformer framework. This enables the model to pay more attention to uncertain regions, leading to more precise segmentation. Lv et al. [26] proposed a novel COD network LSR to simultaneously localize and segment camouflaged objects and rank them according to their detectability. Fan et al. [27] proposed SINet-V2, which first introduced group reverse attention to address the COD problem, and obtained excellent detection performance by combining the location information provided by the neighbor connection decoder module. Recently, Pang et al. [28] proposed a camouflaged object detection model called ZoomNet, which mimics human behavior (zooming in and out) when observing blurred images, using a scaling strategy to learn mixed-scale semantics through scale integration and hierarchical scale mixing.

### 2.2. Multi-Level Feature Fusion

Multi-level feature fusion strategies have been widely used in detection and segmentation tasks. Integrating various levels of feature information enables the effective extraction of contextual semantic information, which enhances the learning capability of the model. Furthermore, coordinating high-level semantic features with low-level fine details is crucial in camouflaged object detection (COD) tasks. Previous works have proposed different multi-level feature fusion strategies. Some methods [29,30,31,32] connect features of the corresponding level in the encoder to the decoder through the transport layer. Since single-level features can only characterize information at a specific scale, this top-down connectivity greatly diminishes the ability to characterize details in low-level features. Each level of features contains rich information, and in order to retain as much information as possible, Refs. [33,34,35] combined features from multiple levels in a fully connected or heuristic manner. However, the extensive integration of cross-scale information tends to lead to high computational costs and a lot of noise, thus reducing the model’s performance. Xia et al. [36] proposed an aggregated interaction strategy with adjacent three-level features as input, fed into each of the three branches, and the information from other branches is flexibly integrated between each branch through interactive learning. The method makes better use of multi-level features, avoids the interference caused by resolution differences in feature fusion, and effectively integrates contextual information from adjacent resolutions. Zhou et al. [24] designed a cross-level fusion and propagation module. It first fuses cross-level features through a series of convolutional layers and residual connections, and then the feature propagation part allows the decoder to obtain more effective features from the encoder to improve the detection performance by weighing the contributions of features from the encoder and decoder. Moreover, we consider the scale variation in the adjacent tertiary features, using the fine detail information in the low-level high-resolution features and the rich semantic information in the high-level low-resolution features as a complement to provide local and global information for the current-level features. In this way, the extraction and fusion of contextual semantic information of multi-level features are facilitated, thus providing rich feature representations for the decoder.

### 2.3. Boundary-Aware Learning

Edge information is increasingly used as auxiliary information to refine object segmentation boundaries, resulting in more accurate segmentation results. Ding et al. [37] suggested learning edges as additional semantic classes to enable the network to learn the boundary layout of scene segmentation effectively. Zhao et al. [3] considered the complementarity between salient edge information and salient object information, and modeled both in the network. By doing so, they fully utilized the salient edge information to achieve more effective object segmentation. Zhu et al. [38] attempted to integrate the boundary information into the feature space using multi-level features of the encoder to enhance the sensitivity of the model to the boundary. Zhou et al. [24] considered that only low-level features contain sufficient boundary information; a boundary guidance module was designed to explicitly model the boundary information by inputting the two lowest-level features from the encoder that contained rich edge details. This module aids in the localization of camouflaged objects and the refinement of edges. Unlike the above methods, our proposed method considers that high-level semantic information can guide the model to filter the edge noise. Therefore, we propose an edge guidance module that explicitly generates the edges of the camouflaged object by combining high-level semantic information and low-level detail information and guides the refinement of camouflaged object edges by embedding the generated edge semantic features into the model.

## 3. Proposed Method

**Figure 1:**
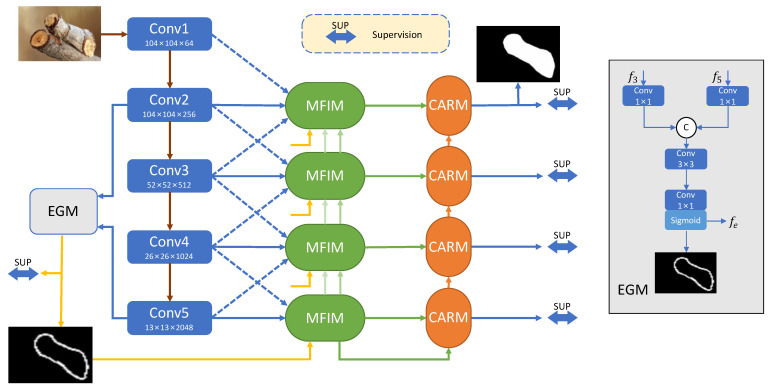
The whole pipeline of the proposed multi-level feature integration network (MFNet), consisting of three main components, i.e., edge guidance module (EGM), multi-level feature integration module (MFIM), and context aggregation refinement module (CARM). Please refer to Section 3 for details.

The overall framework of the proposed MFNet is shown in Figure 1, consisting of three key components: the edge guidance module, multi-level feature integration module, and context aggregation refinement module. Specifically, we use the pre-trained Res2Net-50 [39] as the backbone to extract multi-level features from an input image $I\in R^{H\times W\times 3}$, resulting in a set of features $f_{i},i\in \left\{1,2,3,4,5\right\}$. The resolution of $f_{i}$ is $H/2^{i+1}\times W/2^{i+1},i\in \left\{1,2,3,4,5\right\}$. Next, we propose the EGM, which uses high-level features $f_{5}$ and low-level features $f_{3}$ to model the edge information $f_{e}$ associated with the camouflaged object and obtain the object-related edge semantics. Then, the proposed MFIM integrates multi-level features and edge cues, leveraging high-level semantic information and low-level detail cues to guide the extraction of global and local information by the current layer features, facilitating feature learning and enhancing the boundary representation. Subsequently, the aggregated features are fed into the proposed CARM to effectively integrate cross-level features in a top-down manner, refining the camouflaged object detection results. Finally, we employ a multi-level supervision strategy to improve the COD performance. We will describe the three key modules mentioned above in detail in the following.

### 3.1. Edge Generation Module

Complete edge information is crucial for object localization and segmentation. However, in contrast to the method used in [24], which relies solely on low-level features to obtain edge cues, we argue that low-level features contain many irrelevant non-object edge details. Therefore, it is necessary to utilize the rich semantic information in high-level features to guide the generation of object edge features. For this purpose, we design the edge guidance module (EGM) to explicitly model the edges of camouflaged objects by combining high-level features $f_{5}$ and low-level features $f_{3}$. As shown in Figure 1, when the features enter the EGM, two 1 × 1 convolutional layers are first used to reduce the channels. The features are then integrated by concatenation, and the integrated features are fed to a 3 × 3 convolutional layer to obtain the fused feature representation. Finally, the fused features are fed into a 1 × 1 convolutional layer and a Sigmoid function to obtain the final edge prediction map.

### 3.2. Multi-Level Feature Integration Module

**Figure 2:**
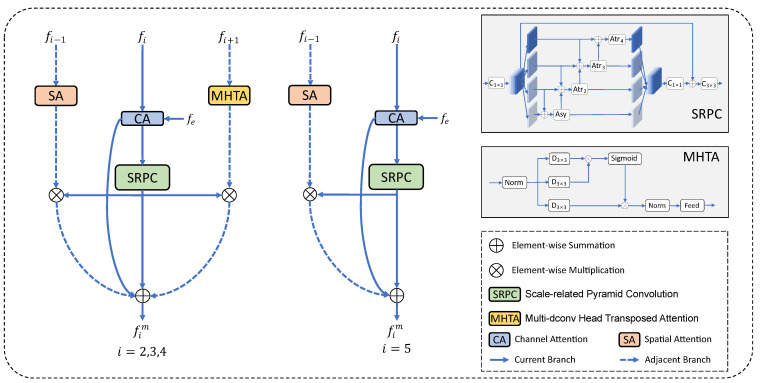
Detailed architecture of the multi-level feature integration module (MFIM). $f^{e}$ is the output feature of EGM.

In COD tasks, high-level features typically contain rich semantic information, while low-level features contain more detailed local clues. To take both semantic and detailed information into account, we propose the multi-level feature integration module (MFIM). We divide MFIM into two cases: the first case is for $f_{2}$, $f_{3}$, and $f_{4}$, which have two adjacent feature layers, while the other case is for $f_{5}$, which has only one adjacent feature layer because of its location. In order to take full advantage of the multi-layer features and reduce the model parameters, we introduce $f_{1}$ into the network as the adjacent low-level feature of $f_{2}$. The framework of MFIM is illustrated in Figure 2. If we define the MFIM process as F(·), its equation can be described as follows:

(1) $$f_{i}^{m}=\left\{\begin{array}{cc} F(f_{i-1},\;f_{i},\;f_{i+1}), & i=2,3,4\\ F(f_{i-1},\;f_{i}), & i=5 \end{array}\right.$$

where $f_{i}^{m}\in R^{h_{i}\times w_{i}\times c_{i}}$ is the output feature of MFIM, and $f_{i-1}$, $f_{i}$ and $f_{i+1}$ are the adjacent low-level features, current-level features, and the adjacent higher-level feature, respectively.

In practice, we design three branches (i.e., one current branch and two adjacent branches) in MFIM. The current branch introduces edge cues through a channel attention module and captures rich contextual information by using atrous convolution and asymmetric convolution with different dilation rates in parallel; for the detailed flow, see Section 3.2.1. The two adjacent branches are the adjacent low-level feature branch and the adjacent high-level feature branch. The adjacent low-level feature branch extracts local detail information via the spatial attention module, while the adjacent high-level feature branch extracts global semantic information via the self-attention mechanism; for details, refer to Section 3.2.2. The features of the three branches are integrated via element-by-element addition operation; for details, refer to Section 3.2.3.

#### 3.2.1. Current Branch

The current branch performs two main operations to process feature $f_{i}$. Firstly, we incorporate the edge cue $f_{e}$ into the network using a channel attention (CA) module [40], which selectively amplifies or suppresses informative channels in feature maps to explore cross-channel interactions and extract the critical information between channels. This process can be formulated as follows:

(2) $$f_{i}^{ca}=CA\left(f_{i},\;f_{e}\right),$$

where $f_{i}^{ca}$ is the output feature of CA, and $f_{e}$ is the edge feature. Then, we utilize the scale-related pyramid convolution (SRPC) module to more effectively combine multi-scale information. This process can be expressed as follows:

(3) $$f_{i}^{s}=SRPC\left(f_{i}^{ca}\right),$$

where $f_{i}^{s}$ is the output feature of the SRPC module. Our proposed SRPC module is dedicated to multi-scale feature learning and integration. Unlike the global context module in BBSNet [41], which independently extracts information at different scales through separate branches, the SRPC module refers to [42], fully considers the cross-scale interaction between adjacent branches, and increases the feature scale diversity through asymmetric convolution and atrous convolution. Specifically, we take the output feature $f_{i}^{k}$ of $f_{i}$ after the 1 × 1 convolutional layer as an example. We divide $f_{i}^{k}$ uniformly into four feature maps along the channel dimension ($f_{i}^{k_{1}}$, $f_{i}^{k_{2}}$, $f_{i}^{k_{3}}$, $f_{i}^{k_{4}}$) for multi-scale learning. This process fuses the features of adjacent branches and obtains multi-scale contextual features via a series of atrous convolutional layers and asymmetric convolutional layers. This process can be formulated as follows:

(4) $$f_{i}^{k_{j}^{\prime }}=\left\{\begin{array}{cc} Conv_{3}^{a}\left(f_{i}^{k_{j}}⊕f_{i}^{k_{j+1}}\right), & j=1\\ Conv_{3}^{n_{j}}\left(f_{i}^{k_{j-1}^{\prime }}⊕f_{i}^{k_{j}}⊕f_{i}^{k_{j+1}}\right), & j=2,3\\ Conv_{3}^{n_{j}}\left(f_{i}^{k{’}_{j-1}}⊕f_{i}^{k_{j}}\right), & j=4 \end{array}\right.$$

where ⊕ is the element-wise summation, $Conv_{3}^{a}$ is a 3 × 3 asymmetric convolutional layer, and $Conv_{3}^{n_{j}}$ is a 3 × 3 atrous convolutional layer with a dilation rate of $n_{j}$. Referring to EDN [42], we set $n_{j}\in \left\{2,3,4\right\}$. Finally, the features ${f_{i}}^{k_{j}^{{}^{\prime }}}$,j∈{1,2,3,4} from the four branches are concatenated and passed through a residual connection, followed by a 3 × 3 convolutional layer. This process can be formulated as follows:

(5) $$f_{i}^{s}=Conv_{3}\left(Concat\left(f_{i}^{k_{1}^{\prime }},f_{i}^{k_{2}^{\prime }},f_{i}^{k_{3}^{\prime }},f_{i}^{k_{4}^{\prime }}\right)\right).$$

where Concat (·) is the concatenation operation, $Conv_{3}$ is a 3 × 3 convolutional layer, and $f_{i}^{s}$ is the output feature of the SRPC module.

#### 3.2.2. Adjacent Branch

Adjacent branches can be divided into two types. One is the adjacent lower-level feature branch, and the lower-level features usually contain more spatial detail information. The spatial attention module [40] focuses on the local information of the feature map by performing a max pooling operation on the feature map in the channel dimension, so we enhance the current feature representation by extracting fine spatial detail information through the spatial attention module, which can be computed as

(6) $$f_{i}^{sa}=SA\left(f_{i-1}\right)⊗f_{i}^{s},$$

where ⊗ is the element-wise multiplication, $f_{i}^{sa}$ is the output feature of the adjacent lower-level feature branch, and $f_{i}^{s}$ is the output feature of the current branch. Another is the adjacent high-level feature branch, where the high-level features contain more contextual semantic information. The multi-dconv head transposed attention (MHTA) module [43] can effectively model long-range dependency relationships, thereby capturing global feature information. Thus, we capture the rich contextual information to enhance the global semantic representation via the MHTA module, which can be computed as

(7) $$f_{i}^{mh}=MHTA\left(f_{i+1}\right)⊗f_{i}^{s},$$

where $f_{i}^{mh}$ is the output feature of the adjacent high-level feature branch.

#### 3.2.3. Branches’ Integration

After being processed by the current and adjacent branches, the features are obtained as $f_{i}^{sa}$, $f_{i}^{s}$, and $f_{i}^{mh}$. Then, they are fused with $f_{i}^{ca}$ (the output feature of CA) by the element-wise summation operation, which can be defined as follows:

(8) $$f_{i}^{m}=\left\{\begin{array}{cc} f_{i}^{sa}⊕f_{i}^{s}⊕f_{i}^{ca}⊕f_{i}^{mh}, & i=2,3,4\\ f_{i}^{s}⊕f_{i}^{ca}⊕f_{i}^{mh}, & i=5 \end{array}\right.$$

where $f_{i}^{m}$ is the output feature of the MFIM.

### 3.3. Context Aggregation Refinement Module

**Figure 3:**
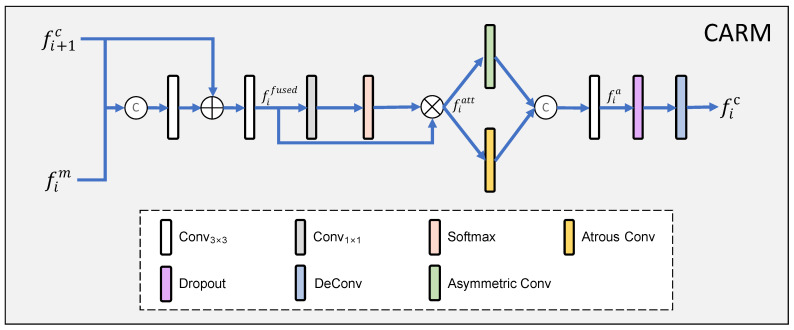
Detailed architecture of the context aggregation refinement module (CARM).

The effective fusion of cross-level features from top to bottom often improves the learning performance. To this end, we propose a context aggregation refinement module (CARM) to improve the detection effect by making full use of the contextual information to refine the features level by level. As shown in Figure 3, for the CARM at the *i*-th (i∈{1,2,3}) stage, it first fuses the output feature of the CARM at the next stage (denoted as $f_{i+1}^{c}$) with the features obtained by the MFIM at the current stage (denoted as $f_{i}^{m}$) through a concatenation operation. The concatenated result is fed to the 3 × 3 convolutional layer and an element-wise summation is performed by adding $f_{i+1}^{c}$. After a 3 × 3 convolution, the high-dimensional features are mapped to the spatial-wise gate through the 1 × 1 convolutional layer, and then the Softmax function is used to obtain the weights to perform element-wise multiplication with the feature $f_{i}^{fused}$ to filter the interference information. We can express this process as follows:

(9) $$f_{i}^{fused}=Conv_{3}\left(Conv_{3}\left(Concat\left(f_{i}^{m},f_{i+1}^{c}\right)\right)⊕f_{i+1}^{c}\right),$$

(10) $$f_{i}^{att}=Softmax\left(Conv_{1}\left(f_{i}^{fused}\right)\right)⊗f_{i}^{fused},$$

where Softmax(·) is the Softmax function and $f_{i}^{att}$ is the output feature of the filtered feature. The atrous convolutional layer and asymmetric convolutional layer can obtain rich contextual semantic information through multi-scale receptive fields. Thus, we further refine the features with atrous convolution and asymmetric convolution, which can be defined as

(11) $$f_{i}^{a}=Conv_{3}\left(Concat\left(Conv_{3}^{asy}\left(f_{i}^{att}\right),Conv_{3}^{atr}\left(f_{i}^{att}\right)\right)\right),$$

(12) $$f_{i}^{c}=DConv_{3}^{de}\left(f_{i}^{a}\right)$$

where $Conv_{3}^{asy}$ is the 3 × 3 atrous convolutional layer with a dilation rate of 3, $Conv_{3}^{atr}$ is the 3 × 3 atrous convolutional layer, $f_{i}^{c}$ is the output feature of the CARM, and $DConv_{3}^{de}$ is a 3 × 3 deconvolution layer followed by a dropout layer. For the last CARM (*i* = 4), the input corresponding to the next CARM is replaced by the output feature of the 4th MFIM.

### 3.4. Loss Function

In pixel-wise segmentation tasks, the binary cross-entropy (BCE) loss and the intersection-over-union (IOU) loss are widely used in conjunction with each other to provide strong constraints on the local pixel and global structure of the object. Inspired by the success of the weighted IOU loss and weighted BCE loss in [4], our detection loss function is defined as

(13) $$L_{det}=L_{BCE}^{w}+L_{IOU}^{w}$$

where $L_{BCE}^{w}$ and $L_{IOU}^{w}$ denote the weighted IOU loss and BCE loss, respectively. $L_{IOU}^{w}$ highlights the importance of hard pixels (a type of pixel that targets difficult samples or misclassified pixels in a pixel-level classification task) by increasing their weights, and $L_{BCE}^{w}$ focuses more on hard pixels rather than treating all pixels equally. Meanwhile, the produced edge map can be measured using the adaptive pixel intensity (API) loss [44], which distinguishes relatively important pixels (pixels that are adjacent to fine or explicit edges) by applying the pixel intensity to the L1 loss. Thus, our total loss can be defined as

(14) $$L_{total}=\sum_{i=1}^{4}L_{det}\left(P_{i},G_{o}\right)+L_{edge}\left(P_{e},G_{e}\right)$$

where $P_{i}$ is the camouflaged object’s predicted map, $G_{o}$ is the ground truth of the camouflaged object, $P_{e}$ is the edge of the camouflaged object’s predicted map, and $G_{e}$ is the ground truth of the edge of the camouflaged object.

## 4. Experiments

### 4.1. Implementation Details

Our model is implemented in PyTorch [45] using Res2Net-50 [39] pre-trained on ImageNet as the backbone. All input images are resized to 416 × 416 and undergo data augmentation via random horizontal flipping. We set the batch size to 16 and employ the Adam optimizer [46]. We initialize the learning rate to 1 × 10${}^{-4}$ and adjust it using a poly strategy with a power of 0.9. The training process, using an NVIDIA RTX 3090 GPU for acceleration, takes around 3.5 h to complete for 60 epochs. The source code and results will be released at https://github.com/WkangLiu/MFNet, accessed on 14 June 2023.

### 4.2. Datasets

We evaluate our model on three popular COD benchmark datasets: CAMO [47], CHAMELEON [48], COD10K [1]. CAMO includes a total of 1250 images, of which 1000 images are used as the training set and 250 images are used as the test set. CHAMELEON contains 76 images collected on the Internet, all of which are used as the test set. COD10K is the largest dataset, containing 5066 images collected on websites classified into 10 super-classes and 78 sub-classes, of which 3040 images are used as the training set and 2026 images are used as the test set.

### 4.3. Evaluation Metrics

We use four widely used evaluation metrics to judge the accuracy of our models, including the structure measure ($S_{\alpha }$) [49], E-measure ($E_{\phi }$) [50], weighted F-measure ($F_{\beta }^{w}$) [51], and mean absolute error (MAE) [14]. In addition, we also provide precision–recall (PR) curves and $F_{\beta }$-threshold ($F_{\beta }$) curves to help to evaluate the model more comprehensively.

#### 4.3.1. Structure Measure ($S_{\alpha }$)

The structure measure is used to measure the structural similarity of region-aware ($S_{o}$) and object-aware ($S_{r}$) aspects, which is defined by

(15) $$S_{\alpha }=\alpha \times S_{o}+\left(1-\alpha \right)\times S_{r},$$

where α is set to 0.5 by default and a higher value of $S_{\alpha }$ leads to the better performance of the models.

#### 4.3.2. E-Measure ($E_{\phi }$)

The E-measure is used to consider the global image-level statistics and the local pixel-matching information, which is defined by

(16) $$E_{\phi }=\frac{1}{W\times H}\sum_{i=1}^{W}\sum_{j=1}^{H}\phi \left(P(x,y),G(x,y)\right),$$

where *W* and *H* are the width and height of the ground truth G, and (x,y) is the coordinate of each pixel in *G*. Symbol ϕ is the enhanced alignment matrix. We obtain a set of $E_{\phi }$ by converting the prediction *P* into a binary mask with a threshold in the range of [0, 255]. A higher value of $E_{\phi }$ leads to the better performance of the models.

#### 4.3.3. Weighted F-Measure ($F_{\beta }^{w}$)

The weighted F-measure is used to consider both precision and recall simultaneously, which is defined by

(17) $$F_{\beta }^{\omega }=\frac{\left(1+\beta^{2}\right)\times Precision^{\omega }\times Recall^{\omega }}{\beta^{2}\times Precision^{\omega }+Recall^{\omega }}$$

where $\mathrm{precision}=\frac{\left|M\cap G\right|}{\left|M\right|}$ and $\mathrm{recall}=\frac{\left|M\cap G\right|}{\left|G\right|}$. Meanwhile, $\beta^{2}$ is set to 0.3 by default and a higher value of $F_{\beta }^{\omega }$ leads to the better performance of the models.

#### 4.3.4. Mean Absolute Error (MAE)

The mean absolute error is used to calculate the average pixel-level relative error between the ground truth and normalized prediction, which is defined by

(18) $$MAE=\frac{1}{W\times H}\sum_{i=1}^{W}\sum_{j=1}^{H}\left|P\left(i,j\right)-G\left(i,j\right)\right|,$$

where *W* and *H* are the width and height of the image. *P* and *G* are the normalized prediction and ground truth, respectively. A smaller value of MAE leads to the better performance of the models.

### 4.4. Comparison with SOTA Methods

We compare the proposed method with 16 state-of-the-art COD baselines: CPD [2], EGNet [3], F3Net [4], UCNet [5], SINet [1], PraNet [6], C2FNet [22], PFNet [21], TINet [17], UGTR [19], R-MGL [18], JCSOD [25], LSR [26], C2FNet-V2 [52], SINet-V2 [27], and BSA-Net [38].

#### 4.4.1. Quantitative Evaluation

**Figure 4:**
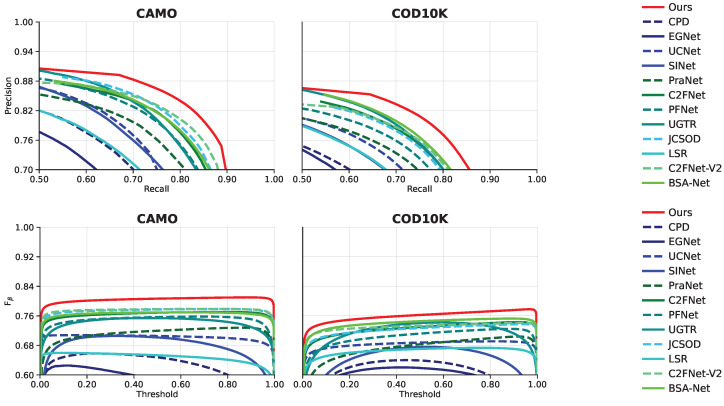
PR and $F_{\beta }$ curves of the proposed method and other SOTA methods on CAMO and COD10K datasets.

Table 1 details the quantitative comparison between our model and the 16 state-of-the-art methods on three benchmark datasets. For a fair comparison, we use the prediction maps provided by the original authors, and if not provided, we directly use their official code and models to compute the missing prediction maps. It can be clearly seen that our network significantly outperforms other advanced models in most evaluation metrics on the three datasets. For instance, on the COD10K dataset, when compared with the second-best method BSA-Net, our method increases $S_{\alpha }$ and $F_{\beta }^{w}$ by 1.9% and 3.9%, respectively, and decreases MAE by 5.9%. On the CAMO dataset, when compared with the second-best method C2FNet-V2, our method increases $S_{\alpha }$ and $F_{\beta }^{w}$ by 3.1% and 4.5%, respectively, and decreases MAE by 13.0%. Overall, our proposed method greatly improves the performance of the SOTA. Figure 4 provides the PR curves and the $F_{\beta }$ curves of our method and other methods on the CAMO and CHAMELEON datasets. The higher the curve in the figure, the better the performance of the model, which further demonstrates the superiority of our method.

#### 4.4.2. Qualitative Evaluation

**Figure 5:**
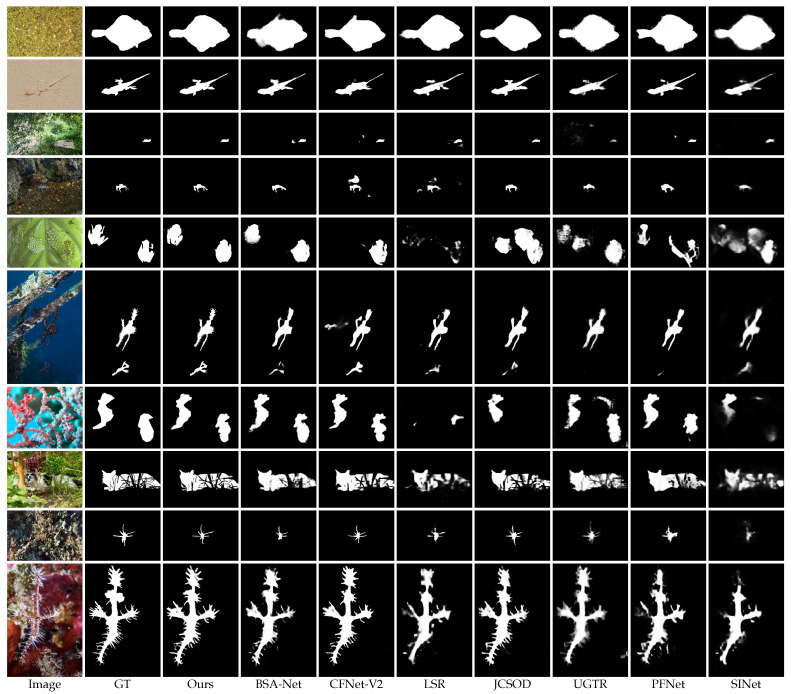
Quantitative evaluation of the proposed MFNet with other SOTA methods (i.e., SINet [1], PFNet [21], UGTR [19], JCSOD [25], LSR [26], C2FNet-V2 [52], and BSA-Net [38]).

In Figure 5, we visualize some challenging scenes and results generated by our method and other SOTA methods. It is not difficult to see that our model can accurately segment objects at different scales, including large objects (Row 1), medium objects (Row 2), small objects (Row 3–4), and multiple objects (Row 5–6). Meanwhile, for objects with high luminance (Row 7), occlusion (Row 8), or abundant edge details (Row 9–10), our method is also able to generate accurate predictions that are very consistent with the ground truth.

### 4.5. Ablation Analysis

#### 4.5.1. Effect of Different Modules

We conduct ablation studies to investigate the effectiveness of the MFIM, CARM, and EGM in our MFNet. Due to the limited number of images in the CHAMELEON test dataset, the poor prediction of individual images may have a large impact on the results, and we only show the experimental results of the models on the CAMO and COD10K datasets. Specifically, the quantitative results of the ablation experiments are summarized in Table 2.

**Figure 6:**
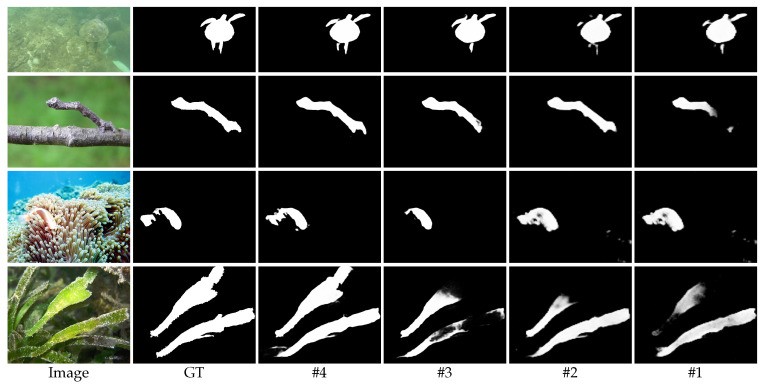
The visual comparison of the detection results obtained by different models in the ablation study. (#1) Baseline, (#2) Baseline+MFIM, (#3) Baseline+MFIM+CARM, (#4) Baseline+MFIM+CARM+EGM (ours).

**Effect of MFIM.** We first adopt a basic model without the MFIM, CARM, and EGM as a baseline (#1). The basic model consists of an encoder–decoder structure, where the encoder uses the backbone network Res2Net-50 [39] and the decoder integrates features layer by layer with a top-down approach. Based on the baseline, by comparing #1 and #2, we find that adding MFIM can significantly improve the detection accuracy. Furthermore, from Figure 6, we can see that the MFIM effectively aggregates multi-scale features. Although a small amount of noise is obtained along with the effective features, it undeniably improves the integrity of camouflaged object detection and enhances the detection accuracy.

**Effect of CARM.** To explore the effectiveness of the CARM, we merged the CARM into #2. As shown in Table 2, compared with #2, the performance of model #3 with the CARM added is significantly improved, which is reflected in the three evaluation metrics for both the CAMO and COD10K datasets. The CARM integrates the rich features output by the MFIM layer by layer in a top-down manner and guides the low-level features with the high-level features, which can help the model to filter out the irrelevant features in the low-level features. This is also verified by the visual comparison results shown in Figure 6. Overall, the inclusion of the CARM further improves the performance of the model.

**Effect of EGM.** After comparing #3 and #4 in Table 2, it can be seen that the EGM can further improve the COD performance, achieving gains of 1.2% for $S_{\alpha }$, 1.8% for $E_{\phi }$, and 7.0% for MAE on the CAMO dataset. In addition, #3 and #4 in Figure 6 can also prove that the addition of edge information makes the edge details of camouflaged object detection clearer, and the ambiguity of the semantics is also effectively alleviated.

#### 4.5.2. Effect of Different Levels of Features as Input in EGM

To verify the importance of high-level features in the EGM to guide edge semantic generation, we designed three variants: (1) low-level features $f_{1}$ and $f_{2}$ as input in the EGM ($f_{1}+f_{2}$), (2) low-level features $f_{1}$ and high-level features $f_{5}$ as input in the EGM ($f_{1}+f_{5}$), and (3) low-level features $f_{2}$ and high-level features $f_{5}$ as input in the EGM ($f_{1}+f_{2}$). We report the quantitative results in Table 3.

We follow FAP-Net [24] and use the low-level features f1 and f2 (#5) as input to the EGM, and the worst results are obtained. Meanwhile, when f1 (#6), f2 (#7), and f3 (#4) are used to explore edges together with f5 to help locate object-related edges, better results are achieved, which proves the effectiveness of using the rich semantic information of high-level features to guide the generation of object edge features. As shown in Table 3, the combination of f3 + f5 (#4) obtains the best performance for camouflaged object detection.

#### 4.5.3. Effect of Different Branches in MFIM

To verify the effectiveness of the two types of branches in the MFIM, we design two variants: (1) removing current branches in the MFIM (without CB) and (2) removing adjacent branches in the MFIM (without AB). We report the quantitative results in Table 4.

The quantitative results show that the performance without CB (#8) and without AB (#9) is worse than with our method (#4), which confirms the effectiveness of the current branch and adjacent branch. Concretely, on the CAMO dataset, the model performance without CB is degraded, e.g., $S_{\alpha }$: 0.824 → 0.765, $E_{\phi }$: 0.883 → 0.802, MAE: 0.067 → 0.090. Comparatively, the model performance without AB declines less significantly on the same dataset, e.g., $S_{\alpha }$: 0.824 → 0.799, $E_{\phi }$: 0.883 → 0.847, MAE: 0.067 → 0.080. A similar situation can be observed for the COD10k dataset. We suggest that the reason is that when the current branch is removed, the local and global information of adjacent branches cannot effectively interact with that of the current branch, which greatly reduces the performance of the model.

**Figure 7:**
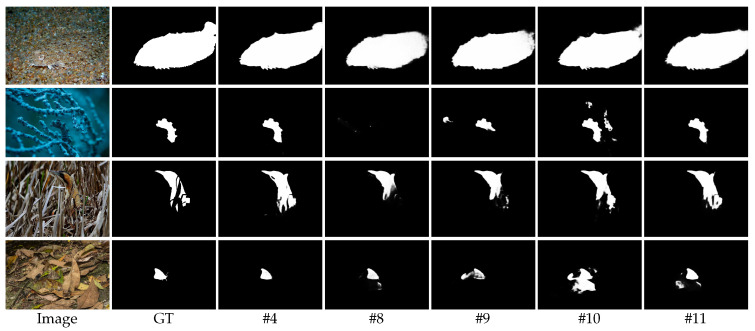
Visual comparison of detection results obtained with the four variants of the MFIM and CARM models in ablation studies. The detection results were obtained (#4) with our method, (#8) without CB, (#9) without AB, (#10) with DC, (#11) with NC.

By observing the visualization results in Figure 7, we can find that both variants are poorly visualized compared to our model. In particular, the visualization without CB (#8) is worse than that without AB (#9), which is consistent with our previous analysis.

#### 4.5.4. Effect of Atrous Convolution and Asymmetric Convolution in CARM

To verify the necessity of atrous convolution and asymmetric convolution in the CARM, we design two variants: (1) replacing atrous convolution and asymmetric convolution with direct connection operations (with DC) and (2) replacing atrous convolution and asymmetric convolution with 3 × 3 convolutional layers (with NC). We report the quantitative results in Table 4.

Our comparison with other ablation analyses reveals that the performance of the two variants differs less from ours. In contrast, atrous convolution and asymmetric convolution (ours) are more conducive to the refinement of camouflaged object detection by the CARM. After reviewing the visualization results in Figure 7, it becomes evident that the three models—with DC (#10), with NC (#11), and ours (#4)—exhibit an incremental improvement in their ability to detect camouflaged objects. In conclusion, the CARM based on atrous convolution and asymmetric convolution can better obtain high-quality contextual semantic information in different sizes and shapes of receptive fields to refine camouflaged objects.

## 5. Downstream Applications

In this section, we apply MFNet to downstream tasks related to COD to evaluate its generalization ability. The datasets used for the three downstream applications are shown in Table 5.

### 5.1. Polyp Segmentation

**Figure 8:**
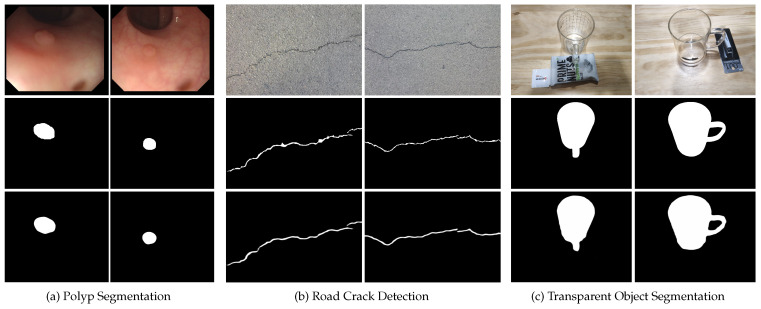
Visualization results of three downstream applications. From top to bottom: image (1st row), ground truth (2nd row), and ours (3rd row).

A polyp is a tumorous lesion that grows in the colon. The accurate segmentation of polyps is crucial in detecting them in colonoscopy images for prompt surgical intervention. In order to evaluate the effectiveness of our method in polyp segmentation, we followed the same benchmark protocol as [6], retrained our MFNet on the KvasirSEG [53] and CVC-ClinicDB [54] datasets, and tested it on the CVC-300 dataset. Figure 8a illustrates the visual results generated by our MFNet.

### 5.2. Defect Detection

Defect detection is an essential process in industrial production to ensure the quality of products. We demonstrate the effectiveness of MFNet in defect detection tasks by taking road crack detection as an example. We retrain our MFNet on the widely used CrackForest [55] dataset, using 60% of the samples for training and 40% for testing. Figure 8b presents the visual results of our approach.

### 5.3. Transparent Object Segmentation

In daily life and industrial production, robots and drones need to accurately identify transparent objects (such as glass, windows, etc.) that are not easily visible, in order to avoid accidents. We further investigate the effectiveness of MFNet in transparent object segmentation tasks. For convenience, we reorganize the annotations of the Trans10K [56] dataset from instance-level to object-level for training purposes. The visual results presented in Figure 8c further demonstrate the generalization ability of MFNet.

## 6. Conclusions

In this paper, we propose a novel multi-level feature integration network (MFNet) for the COD task. We first explicitly model edges with the proposed EGM and use the obtained edge information to guide the network to refine the camouflaged objects’ edges. Secondly, we propose the MFIM to effectively integrate the complete contextual semantic information using the strong correlation of features in adjacent layers. Finally, we propose the CARM to effectively aggregate and refine the cross-layer features to obtain clear prediction maps. Through extensive experiments, we prove that our MFNet outperforms other state-of-the-art COD methods and exhibits excellent detection performance.

## Figures and Tables

**Table 1:** Quantitative evaluation results on three benchmark datasets regarding S-measure, E-measure, weighted F-measure, and MAE scores. The best results are highlighted in bold. “↑” and “↓” indicate that larger or smaller is better.

Method	Year	CAMO				CHAMELEON				COD10K			
		$S_{\alpha }$↑	$E_{\phi }$↑	$F_{\beta }^{w}$↑	MAE↓	$S_{\alpha }$↑	$E_{\phi }$↑	$F_{\beta }^{w}$↑	MAE↓	$S_{\alpha }$↑	$E_{\phi }$↑	$F_{\beta }^{w}$↑	MAE↓
CPD	2019	0.716	0.796	0.658	0.113	0.857	0.898	0.813	0.048	0.750	0.853	0.640	0.053
EGNet	2019	0.662	0.766	0.612	0.124	0.848	0.831	0.676	0.050	0.737	0.810	0.608	0.056
F3Net	2020	0.711	0.780	0.630	0.109	0.848	0.917	0.798	0.047	0.739	0.819	0.609	0.051
UCNet	2020	0.739	0.787	0.700	0.095	0.880	0.930	0.836	0.036	0.776	0.857	0.681	0.042
SINet	2020	0.745	0.829	0.644	0.092	0.872	0.946	0.806	0.034	0.776	0.864	0.631	0.043
PraNet	2020	0.769	0.837	0.663	0.094	0.860	0.907	0.763	0.044	0.789	0.861	0.629	0.045
C2FNet	2021	0.796	0.864	0.719	0.080	0.888	0.935	0.828	0.032	0.813	0.890	0.686	0.036
PFNet	2021	0.782	0.842	0.695	0.085	0.882	0.931	0.810	0.033	0.800	0.877	0.660	0.040
TINet	2021	0.781	0.848	0.678	0.087	0.874	0.916	0.783	0.038	0.793	0.861	0.635	0.042
UGTR	2021	0.784	0.851	0.684	0.086	0.888	0.940	0.794	0.031	0.818	0.853	0.667	0.035
R-MGL	2021	0.775	0.847	0.673	0.088	0.893	0.923	0.813	0.030	0.814	0.852	0.666	0.035
JCSOD	2021	0.800	0.873	0.728	0.073	0.894	0.943	0.848	0.030	0.809	0.884	0.684	0.035
LSR	2021	0.787	0.854	0.696	0.080	0.893	0.938	0.839	0.033	0.804	0.880	0.673	0.037
C2FNet-V2	2022	0.799	0.859	0.730	0.077	0.893	0.947	0.845	0.028	0.811	0.891	0.691	0.036
SINet-V2	2022	0.820	0.882	0.743	0.070	0.888	0.942	0.816	0.030	0.815	0.887	0.680	0.037
BSA-Net	2022	0.796	0.851	0.717	0.079	0.895	0.946	0.841	0.027	0.818	0.891	0.699	0.034
Ours	-	**0.824**	**0.883**	**0.763**	**0.067**	**0.904**	**0.948**	**0.856**	**0.026**	**0.834**	**0.901**	**0.726**	**0.032**

**Table 2:** Ablation analyses of each component on the CAMO and COD10K datasets. Bold: top result. The quantitative evaluation results obtained by (#1) Baseline, (#2) Baseline+MFIM, (#3) Baseline+MFIM+CARM, (#4) Baseline+MFIM+CARM+EGM (ours).

No.	B	MFIM	CARM	EGM	CAMO			COD10K		
					$S_{\alpha }$↑	$E_{\phi }$↑	MAE↓	$S_{\alpha }$↑	$E_{\phi }$↑	MAE↓
**#1**	🗸				0.782	0.813	0.083	0.811	0.859	0.035
**#2**	🗸	🗸			0.803	0.844	0.078	0.821	0.868	0.034
**#3**	🗸	🗸	🗸		0.814	0.867	0.072	**0.834**	0.899	0.033
**#4**	🗸	🗸	🗸	🗸	**0.824**	**0.883**	**0.067**	**0.834**	**0.901**	**0.032**

**Table 3:** Ablation analysis of three variant models modified for EGM on the CAMO and COD10K datasets. Bold: top result. The quantitative evaluation results obtained by (#5) $f_{1}+f_{2}$, (#6) $f_{1}+f_{5}$, (#7) $f_{2}+f_{5}$, (#4) $f_{3}+f_{5}$ (ours).

No.	Models	CAMO			COD10K		
		$S_{\alpha }$↑	$E_{\phi }$↑	MAE↓	$S_{\alpha }$↑	$E_{\phi }$↑	MAE↓
**#5**	** $f_{1}+f_{2}$ **	0.794	0.845	0.079	0.825	0.887	0.033
**#6**	** $f_{1}+f_{5}$ **	0.804	0.848	0.079	0.822	0.876	0.034
**#7**	** $f_{2}+f_{5}$ **	0.817	0.876	0.071	**0.834**	0.898	**0.032**
**#4**	** $f_{3}+f_{5}$ **	**0.824**	**0.883**	**0.067**	**0.834**	**0.901**	**0.032**

**Table 4:** Ablation analysis of four variant models modified for MFIM and CARM on the CAMO and COD10K datasets. Bold: top result. The quantitative evaluation results obtained (#4) with our method, (#8) without CB, (#9) without AB, (#10) with DC, (#11) with NC.

No.	Models	CAMO			COD10K		
		$S_{\alpha }$↑	$E_{\phi }$↑	MAE↓	$S_{\alpha }$↑	$E_{\phi }$↑	MAE↓
**#8**	**Without CB**	0.765	0.802	0.090	0.815	0.863	0.035
**#9**	**Without AB**	0.799	0.847	0.080	0.823	0.883	0.034
**#10**	**With DC**	0.820	0.881	0.070	0.830	0.897	0.034
**#11**	**With NC**	**0.825**	0.880	0.068	0.832	0.897	0.034
**#4**	**Ours**	0.824	**0.883**	**0.067**	**0.834**	**0.901**	**0.032**

**Table 5:** Three downstream applications and the datasets used for each.

Downstream Application	Dataset Used
Polyp Segmentation	KvasirSEG Dataset [53], CVC-ClinicDB Dataset [54], and CVC-300 Dataset
Defect Detection	CrackForest Dataset [55]
Transparent Object Segmentation	Trans10K Dataset [56]

## Data Availability

Not applicable.
